# Radiation Enhancer Effect of Platinum Nanoparticles in Breast Cancer Cell Lines: In Vitro and In Silico Analyses

**DOI:** 10.3390/ijms22094436

**Published:** 2021-04-23

**Authors:** Marie Hullo, Romain Grall, Yann Perrot, Cécile Mathé, Véronique Ménard, Xiaomin Yang, Sandrine Lacombe, Erika Porcel, Carmen Villagrasa, Sylvie Chevillard, Emmanuelle Bourneuf

**Affiliations:** 1 Commissariat à l’Energie Atomique et aux Energies Alternatives (CEA), Fundamental Research Division (DRF), François Jacob Institute (IBFJ), Institute of Molecular and Cellular Radiobiology (IRCM), Laboratoire de Cancérologie Expérimentale (LCE), University Paris Saclay, Route du Panorama, CEDEX, 92265 Fontenay-aux-Roses, France; marie.hullo@cea.fr (M.H.); romain.grall@cea.fr (R.G.); cecile.mathe@cea.fr (C.M.); veronique.menard@cea.fr (V.M.); sylvie.chevillard@cea.fr (S.C.); 2 IRSN, Institut de Radioprotection et de Sûreté Nucléaire, BP17, 92962 Fontenay-aux-Roses, France; yann.perrot@irsn.fr (Y.P.); carmen.villagrasa@irsn.fr (C.V.); 3 Institut des Sciences Moléculaires d’Orsay, Université Paris Saclay, CNRS UMR 8214, 91405 Orsay, France; xiaomin.yang@universite-paris-saclay.fr (X.Y.); sandrine.lacombe@universite-paris-saclay.fr (S.L.); erika.porcel@universite-paris-saclay.fr (E.P.)

**Keywords:** platinum nanoparticle, ionizing radiation, dose enhancement effect, radiation enhancement effect, radiation sensitivity, radiation resistance

## Abstract

High-Z metallic nanoparticles (NPs) are new players in the therapeutic arsenal against cancer, especially radioresistant cells. Indeed, the presence of these NPs inside malignant cells is believed to enhance the effect of ionizing radiation by locally increasing the dose deposition. In this context, the potential of platinum nanoparticles (PtNPs) as radiosensitizers was investigated in two breast cancer cell lines, T47D and MDA-MB-231, showing a different radiation sensitivity. PtNPs were internalized in the two cell lines and localized in lysosomes and multivesicular bodies. Analyses of cell responses in terms of clonogenicity, survival, mortality, cell-cycle distribution, oxidative stress, and DNA double-strand breaks did not reveal any significant enhancement effect when cells were pre-exposed to PtNPs before being irradiated, as compared to radiation alone. This result is different from that reported in a previous study performed, under the same conditions, on cervical cancer HeLa cells. This shows that the efficacy of radio-enhancement is strongly cell-type-dependent. Simulation of the early stage ionization processes, taking into account the irradiation characteristics and realistic physical parameters in the biological sample, indicated that PtNPs could weakly increase the dose deposition (by 3%) in the immediate vicinity of the nanoparticles. Some features that are potentially responsible for the biological effect could not be taken into account in the simulation. Thus, chemical and biological effects could explain this discrepancy. For instance, we showed that, in these breast cancer cell lines, PtNPs exhibited ambivalent redox properties, with an antioxidant potential which could counteract the radio-enhancement effect. This work shows that the efficacy of PtNPs for enhancing radiation effects is strongly cell-dependent and that no effect is observed in the case of the breast cancer cell lines T47D and MDA-MB-231. Thus, more extensive experiments using other relevant biological models are needed in order to evaluate such combined strategies, since several clinical trials have already demonstrated the success of combining nanoagents with radiotherapy in the treatment of a range of tumor types.

## 1. Introduction

Among the therapeutic strategies available to combat solid tumors, radiotherapy (RT) is used in more than half of cases. RT consists of the delivery of ionizing radiation to the cancer cells, leading to direct and indirect DNA damage. Indeed, ionization cannot only directly induce nucleic acid strand breaks, but it can also produce free radicals by water radiolysis. Thus, these species contribute to DNA damage, leading to cell death, depending on the cancer cell radiation sensitivity [1]. Indeed, tumors are very heterogeneous entities and contain variable proportions of cells resistant to treatments. Radiation resistance, which is responsible for many treatment failures and cancer relapses, can be attributed to innate mechanisms, such as genetic and mutation specificities of cancer cells, and/or can be acquired by cancer cells during the treatment. Research on radiotherapy currently focuses on increasing the dose and dose rate delivered to a tumor with a better ballistic for preserving healthy tissues. Concerning the latter point, the use of metallic high-Z nanoparticles (NPs) seems very promising for enhancing the radiation effect within a tumor, as the photoelectric interaction cross-section is dependent on Z^5^. Once they are in the cells and upon irradiation, these metallic NPs emit showers of secondary electrons, which are added to those generated by the ionization of water molecules in the biological medium, thus intensifying local energy deposits and intracellular ROS production. In a mouse mammary carcinoma model, Hainfeld et al. [2] showed a clear radio-sensitization effect of gold nanoparticles delivered to tumors before irradiation. This first in vivo demonstration paved the way for a growing number of publications, which indicates the potential of high-Z nanoparticles in terms of enhancing the ionizing radiation effect. In this connection, gold, gadolinium, and hafnium nanoparticles are mainly studied [1].

While platinum (Z = 78) nanoparticles also fulfill the necessary criteria for achieving a radiation enhancement effect, the literature is still scarce. Most studies have focused on very small Pt nanoparticles [3,4,5,6,7,8], while few have focused on larger and more complex structures, such as nanoflowers [9], nanodendrites [10,11], and bimetallic nanoparticles [12].

This study is the first to explore the possible radio-sensitization of human breast cancer cell lines by PtNPs. More than 50% of malignant breast tumors are treated with radiotherapy, and despite a majority of favorable outcomes, some resistant tumors relapse. In this study, two cell lines were used as models of breast cancer, differing in the level of aggressivity and radiation resistance. Different biological endpoints, such as cell survival/death, proliferation, DNA double-strand breaks, and ROS, were analyzed after the exposure to PtNPs and ionizing radiation. Overall, PtNPs did not provide any enhancer effect in the two breast cancer cell lines in comparison to radiation only. This is consistent with the results obtained through Geant4 modeling, which predict a rather limited effect on physical dose deposition and only in the immediate vicinity of PtNPs. Under our experimental conditions, this enhanced physical step cannot generate direct damage to DNA and is not believed to generate major biological responses.

## 2. Results

### 2.1. Nanoparticle Characterization

As recommended, PtNPs were suspended in water and in a culture medium and were characterized by DLS measurements in either water or the culture medium. The Z-average values in water and DMEM are 25.8 and 19.7 nm, respectively. The measured zeta-potential is −16 mV in water.

### 2.2. Nanoparticle Uptake by Breast Cancer Cells

First, we sought to determine if PtNPs were internalized in the cells and what their intracellular localization was. The PtNPs uptake was characterized after exposure of two different breast cancer cell lines, T47D and MDA-MB-231.

**Figure 1:**
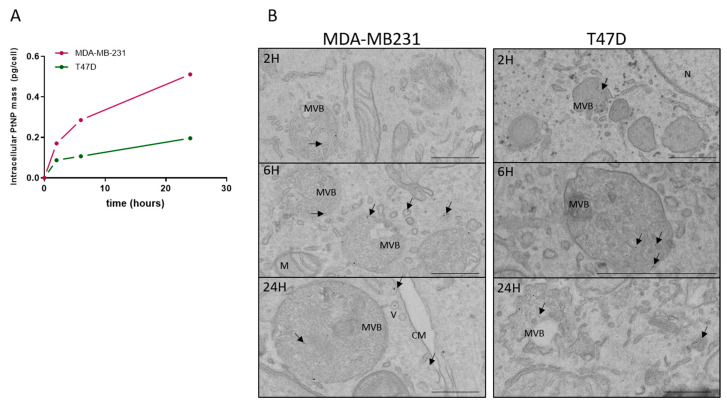
Assessment of the nanoparticle uptake in the MDA-MB-231 and T47D cells. (**A**) Absolute quantification of the intracellular mass of platinum nanoparticles per cell, following an exposure of 2, 6, or 24 h and measured by ICP–MS. (**B**) Representative images obtained by using Transmission Electron Microscopy of the MDA-MB-231 (left) and T47D (right) cells, after being exposed for 2, 6, or 24 h to 0.5 mM PtNPs. Nanoparticles are indicated by arrows. M = mitochondria, MVB = multivesicular body, V = vesicle, CM = cell membrane, N= nucleus. Scale bar = 500 nm.

The ICP-MS absolute quantification of intracellular platinum was performed on cell extracts after 2, 6, or 24 h of exposure. In the two cell lines, a gradual uptake of nanoparticles is observed, with the highest uptake rate within the first two hours of exposure (Figure 1A). Meanwhile, at a slower pace, the intracellular platinum amounts continue to increase until 24 h. Interestingly, at that point, the platinum mass is higher in the MDA-MB-231 cells (0.5 pg/cell) than in the T47D cells (0.2 pg/cell). In order to investigate the intracellular distribution of nanoparticles, Transmission Electronic Microscopy (TEM) was used, taking advantage of the possible visualization of platinum particles without labeling (Figure 1B). Images were obtained after 2, 6, or 24 h of nanoparticle exposure for the MDA-MB-231 and T47D cells. As early as 2 h after the beginning of exposure, nanoparticles (indicated by arrows) can be observed in intracellular vesicles (V) and multivesicular bodies (MVB) or late endosomes. As shown on the picture taken at 24 h on the MDA-MB-231 cells, nanoparticles are internalized in cells within endocytic vesicles and probably transported to endosomes, before being sequestered in MVBs. The number of nanoparticles per cell increases with the time of exposure, confirming what is quantitatively measured by ICP–MS.

### 2.3. In Vitro Radio-Enhancement Effect of PtNPs

**Figure 2:**
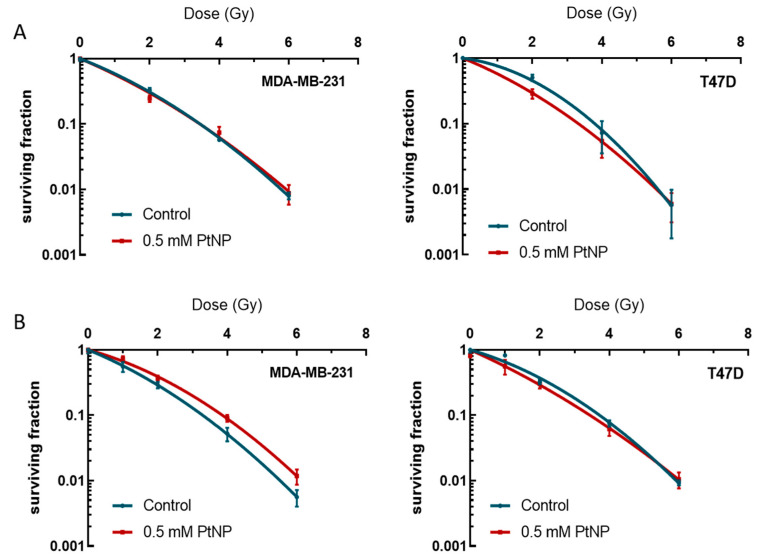
Survival of MDA-MB-231 and T47D cells, as determined by clonogenic assays. The surviving fractions of each cell line and each condition were plotted against the radiation dose. (**A**) Cells were exposed to 0.5 mM PtNPs for 6 h; irradiated at 2, 4, or 6 Gy; and cultured for 14 days (MDA-MB-231, 5 replicates) or 28 days (T47D, two independent sets of 5 replicates). (**B**) Cells were exposed to 0.5 mM PtNPs for 24 h; irradiated at 1, 2, 4, or 6 Gy; and cultured for 14 days (MDA-MB-231, 5 replicates) or 28 days (T47D, 5 replicates).

A clonogenic assay was performed to determine the extent of the radio-sensitization of the breast cancer cell lines by PtNPs, as compared to radiation alone. For each cell type, MDA-MB-231 and T47D, the surviving fraction was assessed following irradiation of cells pre-exposed to 0.5 mM PtNPs or not. The results shown in Figure 2 present the clonogenic survival of cells exposed to PtNPs for 6 h (A) or 24 h (B) in response to different doses of ionizing radiation. The results shown in Figure 2 show that the MDA-MB-231 clonogenic survival after irradiation is similar, irrespective of whether the cells were pre-exposed or not to PtNPs for 6 h. For the 24-h exposure time, the two curves do not superimpose, but surprisingly, the PtNPs-exposed cells show a better survival than the control cells. Concerning the T47D cells, the clonogenic survival is only impaired for the lowest dose of 2 Gy. By increasing the duration of exposure in order to optimize the intracellular concentration of PtNPs, the T47D cells benefit from no enhancing effect. Therefore, except for the 2 Gy irradiation after 6 h of exposure of the T47D cells, the intracellular presence of PtNPs does not modify the clonogenic survival of these cells after irradiation.

**Figure 3:**
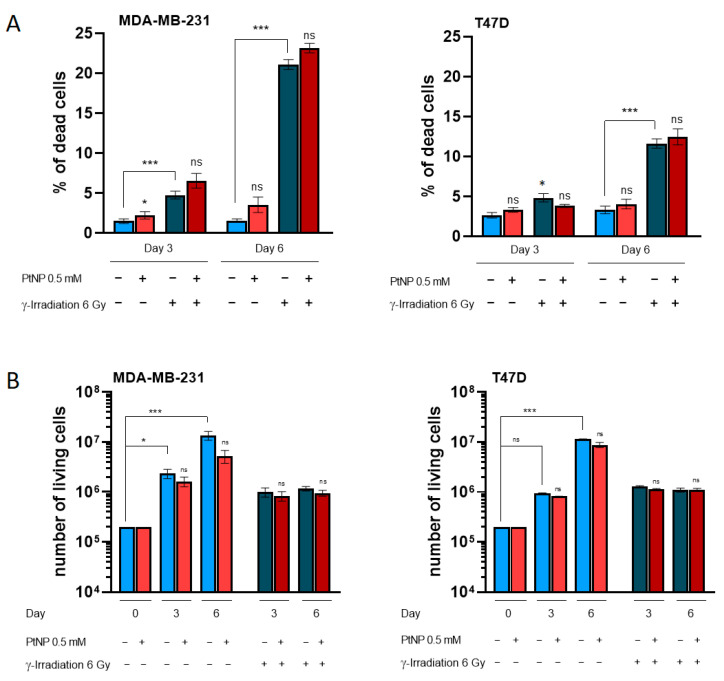
Quantification of cell mortality and survival after the irradiation of cells pretreated with PtNPs or not. (**A**) Percentage of dead cells measured by using the PI exclusion method. Cells were irradiated at 6 Gy after being exposed to 0.5 mM NPs or not, and the mortality was evaluated after 3 or 6 days of culture. The experiment was conducted on two independent sets of triplicates for each time point for the MDA-MB-231 cells and on one triplicate for day 3 and two independent sets of triplicates for day 6 for the T47D cells. (**B**) Measurement of the proliferation by counting the absolute living cell numbers. Cells were irradiated at 6 Gy after being exposed to 0.5 mM NPs or not. Cell counts were assessed by flow cytometry, after 3 or 6 days of culture post-exposure. The experiment was conducted on two independent sets of triplicates for each time point for the MDA-MB-231 cells and on one triplicate for day 3 and two independent sets of triplicates for day 6 for the T47D cells. The statistical significance was determined by using the Kruskal–Wallis test. Note: ns, non-significant; * *p* < 0.05 and *** *p* < 0.001.

We then investigated in more detail if exposure to PtNPs had an effect on cell proliferation and mortality (Figure 3). While barely significant, the exposure of MDA-MB-231 cells to PtNPs induces a slight increase in the percentage of dead cells (Figure 3A, *p* < 0.05 at day 3 and non-significant at day 6). Three days post-irradiation, the MDA-MB-231 cell death increases (*p* < 0.001), with no difference between PtNPs-exposed cells or not. The difference between the control and γ-irradiated cells is even larger after 6 days of culturing, reaching more than 20% of cells. Again, the presence of PtNPs prior to irradiation does not enhance the MDA-MB-231 mortality. In the T47D cells, after 3 days of culturing, the only significant difference corresponds to a faint increase in the percentage of dead cells between the control and irradiated cells (*p* < 0.05). At day 6, however, the mortality reaches more than 10% of cells, compared to the control cells, but the exposure with PtNPs has no effect. Therefore, in the two cell lines, the pre-exposure of cells with PtNPs does not enhance mortality, compared to IR only.

In order to evaluate the cell survival, cells were exposed to 0.5 mM PtNPs for 6 h and/or irradiated at 6 Gy (Figure 3B). An increase in the cell number is observed at days 3 and 6, consistent with the standard proliferation rate of the two cell lines. The 6-h exposure with nanoparticles did not affect the proliferation, since the living cell numbers are equivalent in exposed versus non-exposed cultures. In contrast, irradiation induced cell-proliferation arrest, whether in the presence of PtNPs or not. At day 3, the number of cells is equivalent between irradiated and non-irradiated cells in the MDA-MB-231 and T47D cells. However, at day 6, the number remains similar, which indicates a slower proliferation rate and increase in mortality.

**Figure 4:**
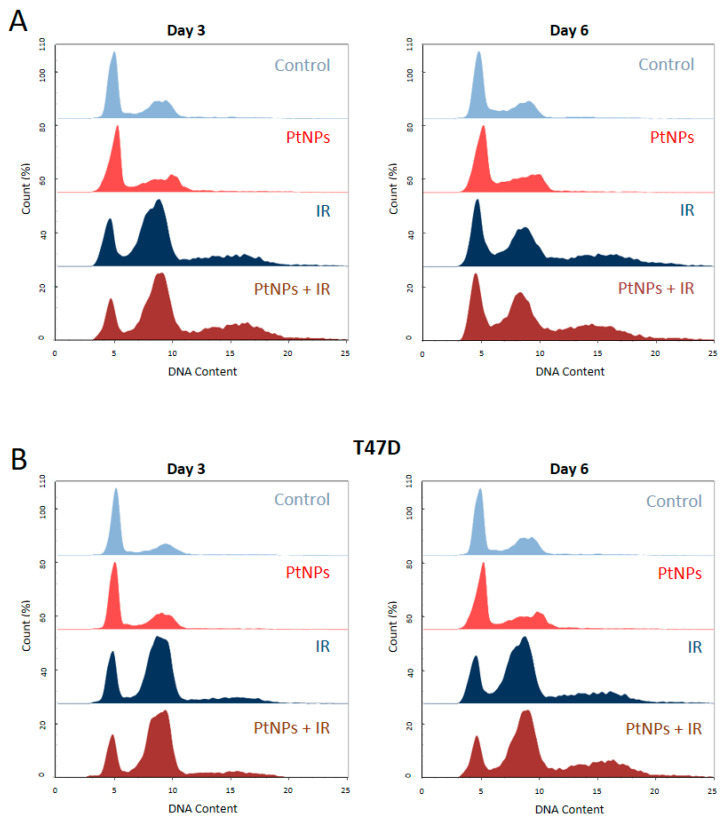
Cell-cycle analysis of 6 Gy-irradiated breast cancer cells pretreated or not with 0.5 mM PtNPs. (**A**) Cell-cycle distribution of the MDA-MB-231 cells exposed to 0.5 mM PtNPs and/or 6 Gy irradiation. Cells were harvested at day 3 (*n* = 3) or at day 6 (*n* = 3) after the exposure and/or irradiation. (**B**) Cell-cycle distribution of the T47D cells exposed to PtNPs and/or 6 Gy irradiation. Cells were harvested at day 3 (*n* = 1) or at day 6 (*n* = 2) after the exposure and/or irradiation.

The distribution of cells within all cell-cycle phases was assessed by quantifying the propidium iodide incorporation by flow cytometry (Figure 4). Cells were irradiated at 6 Gy, after being exposed to 0.5 mM PtNPs or not. After 3 or 6 days of culture, the cells were harvested, and the cell-cycle distribution was determined for each condition. The exposure to PtNPs alone did not induce any change in the cell-cycle distribution for both cell lines. The 6 Gy–irradiation led to an expected shift of cells towards a G2/M blockade and a hyperploidy. When the cells were exposed to nanoparticles before irradiation, there were no significant changes in the patterns observed.

**Figure 5:**
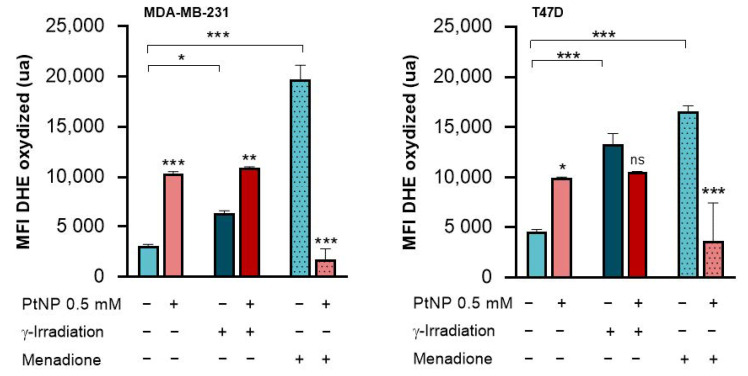
ROS quantification in breast cancer cells exposed to 0.5 mM PtNPs or not and further irradiated or treated with menadione. The fluorescence emitted by the oxidized DHE probe was measured for the MDA-MB-231 and T47D cell cultures, after exposure to PtNPs and/or irradiation with 6 Gy. The two last columns of each graph represent the fluorescent signals obtained with an incubation with menadione, an inducer of oxidative stress. For each condition, 4 replicates were analyzed. Note: ns = non-significant; * *p* < 0.05, ** *p* < 0.01, and *** *p* < 0.001.

The production of ROS was then investigated by measuring the DHE probe’s fluorescence in the MDA-MB-231 and T47D cells exposed to PtNPs and/or irradiated at 6 Gy. As can be observed in Figure 5, PtNPs alone increased the ROS in the two cell lines at rather equivalent levels, and, as expected, γ-irradiation alone also increased the ROS quantity in the two cell lines. However, when cells are exposed to PtNPs prior to irradiation, the levels of ROS reach, in the two cell lines, exactly the same levels as those obtained after exposure to PtNPs alone, indicating that after the double-exposure, we cannot detect any additional ROS within the cells. To understand the latter point, we decided to treat cells with menadione to induce, first, a stronger oxidative stress and, second, a distinct oxidative stress, as compared to radiation (Figure 5). Indeed, menadione mainly induces superoxide anion and then hydrogen peroxide, while radiation first induces peroxide anions and then hydroxyl radicals. When cells are treated with menadione alone, the quantity of oxidized DHE probes is increased, while when cells are pre-exposed to PtNPs, the level of oxidation remains equivalent to that of the untreated control cells (Figure 5). These results could indicate that PtNPs have ROS scavenger capacities.

**Figure 6:**
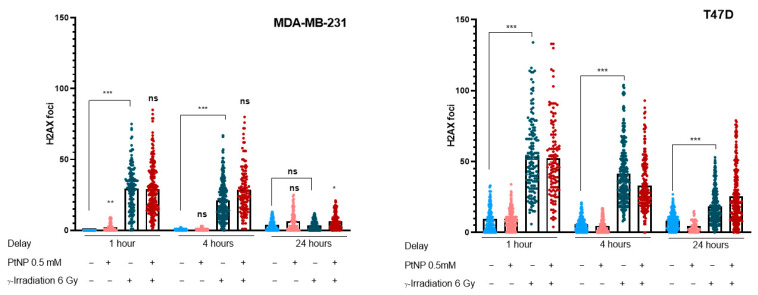
Double-strand DNA breaks counted after the 6 Gy irradiation of breast cancer cells pretreated with 0.5 mM PtNPs or not. The DSB foci were detected by labeling with H2A.X antibody and counted 1, 4, or 24 h after irradiation. For each condition tested, a minimum of 100 nuclei were investigated. An unpaired t-test was used to identify outliers, and a Kruskal–Wallis multiple comparison was conducted to determine the significance of the findings. Note: ns = non-significant; * *p* < 0.05, ** *p* < 0.01, and *** *p* < 0.001.

The number of DNA double-strand breaks (DSBs) reflects treatment-induced genotoxicity. Since DNA breaks specifically induce phosphorylation of the histone H2A.X protein, labeling the nuclei with an antibody targeting γH2A.X allows for a direct quantification of the number of DSBs. Therefore, the number of γH2A.X foci was reported 1, 4, or 24 h after the exposure to NPs or the irradiation (Figure 6). Interestingly, a 6-h exposure to PtNPs only induces a few DSBs in the MDA-MB-231 cells, compared to the control cells. When irradiated, the cells undergo a burst of DSBs (mean = 30, compared to 0–3 in the controls), with no significant difference when the cells were pretreated with PtNPs. The results are similar after a 1- and 4-h delay. After a 24 h delay, the number of foci in the control cells decreases back to the control level, which is a normal consequence of DNA repair. The cells pre-exposed to PtNPs are still significantly different from the irradiated-only cells, which potentially indicates a repair delay induced by the double treatment. In the T47D cells, the number of foci observed in each condition is higher than that in the MDA-MB-231 cells. This is expected, since it is known that T47D epithelial cells are more prone to genotoxicity than mesenchymal MDA-MB-231 cells. As for the MDA-MB-231 cells, irradiation leads to a dramatic increase in the number of foci, but the exposure to PtNPs does not have an enhancer effect on the number of DNA breaks. DSBs decreased at 24 h, with a slight but not significant difference when cells were pretreated with PtNPs.

### 2.4. Simulation of the Secondary Electron Energy Spectra Generated by PtNPs Irradiation

**Figure 7:**
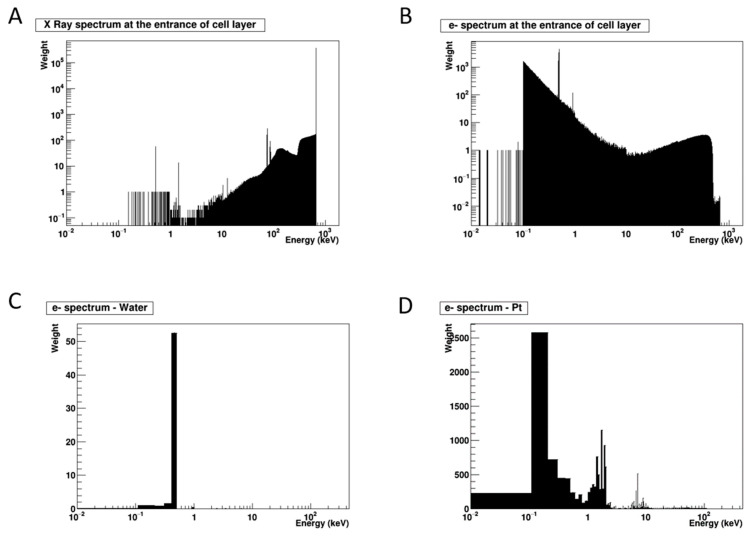
Simulation of the energy spectra at the entrance of the cell layer and released by the nanoparticles. (**A**) Energy spectra of X-rays entering the cell culture. (**B**) Energy spectra of electrons entering the cell culture. (**C**) Energy spectra of the electrons produced in a nanoparticle made of liquid water and Pt (**D**) and escaping the nanoparticle.

**Figure 8:**
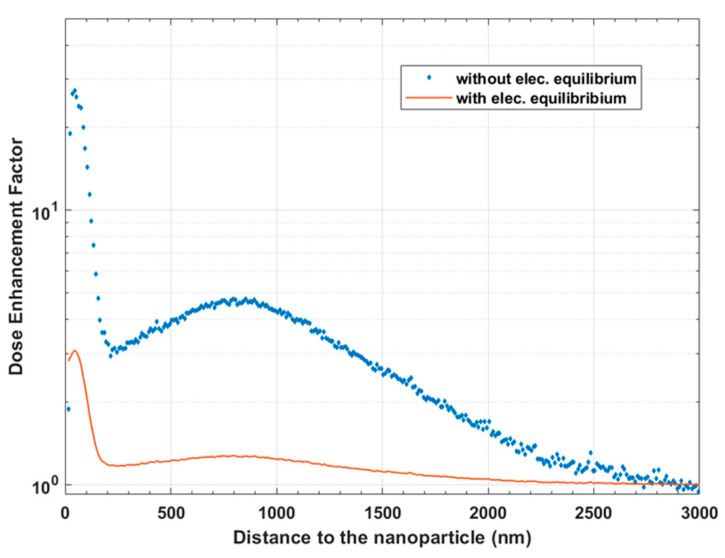
Dose-Enhancement Factor according to the distance to the nanoparticle, considering or not considering the electronic equilibrium.

Beyond long-standing hypotheses, we sought to evaluate the energy of particles reaching the cells in realistic in vitro conditions, as well as the potential increase in energy deposited within a cell after irradiating a PtNP. The first step of the study consisted in simulating the primary and secondary particles obtained by irradiation of the cell culture, and the second step consisted in calculating the energy released by the plurienergetic spectra hitting the PtNP, as compared to water as a control (Figure 7 and Figure 8). Figure 7A shows the X-ray energy spectra interacting with the cell layer. According to the X-ray spectra (Figure 7A), most of the photons are primary photons, which transferred very small amounts of energy. This is due to the configuration of the experimental setup and the use of 137Cs as a source. As a result, most of the photons have an energy close to their initial energy (662 keV) and interact mainly by Compton effect inside the cell. A very small number of photons have energies that will lead to the desired photoelectric effect in the nanoparticle (K-edge of Pt is 78.4 keV). In that case, electrons of different energy levels are generated (Figure 7B) and deposit their energy into the cells, leading to a breakage of the chemical bonds of biomolecules. The Dose-Enhancement Factor (DEF) reflects the additional contribution of irradiated PtNP to the secondary electron emission, as compared to the secondary electron emission after water irradiation alone. Thus, when calculating the DEF, this energy deposition (Figure 7B) must be taken into account, as there is a risk of overestimating this factor if it is not considered [13]. All the particles described above—both primary and secondary electrons—interact with the nanoparticles, and the resulting additional electrons that escape from the nanoparticle are thus those that will have a potential enhancement effect. The energy distributions are shown in Figure 8. Because of its chemical composition (Zeff = 7.2), very few interactions occurred in water (Figure 7C). Due to the high atomic number of platinum, the interactions are much more frequent, and thus it induces a more efficient production of electrons; however, some are absorbed by the nanoparticle itself and never interact with the cell components (Figure 7D). Thus, once the ionizing radiation interacts with the PtNP, a local increase in energy deposition is expected, which will be accompanied by an increase in the number of radical species resulting from water radiolysis. However, it should be noted that most of the electrons escaping from the nanoparticle have an energy of less than 3 keV, which represents a range of less than 300 nm in liquid water.

Figure 8 shows the evolution of DEF as a function of the distance to the surface of the nanoparticle. The results that take into consideration the electronic equilibrium and those that do not are both shown. Two peaks are detectable, one at 50 nm, the other at 800 nm, which are due to the components of the spectra of electrons escaping from the nanoparticle, as shown in Figure 7. The main peak, linked to the Auger emission, has a very low range. It has recently been reported [13] that, without taking into account the electron balance, i.e., when the beam of particles arriving in the nanoparticle has a size equivalent to the size of the nanoparticle, the DEF is very largely overestimated. In our study, the overestimation reaches a factor of 10. It is therefore essential to look precisely at how the DEF is calculated. In our case, the calculation at equilibrium shows that the DEF maximum value is 3.1 at 45 nm around the nanoparticle and that the DEF reaches the value of 2 for distances higher than 100 nm. Then, there is a stabilization between 10% and 24% up to 1.5 µM from the nanoparticle. Finally, taking into account an uncertainty of 5% in the calculation, we can say that beyond 1.9 µM, the enhancement effect is no longer appreciable. The DEF prediction, according to the physical parameters in this experimental biological setup, shows that the enhancement effect is localized in the immediate vicinity of the nanoparticle and that an effect at a larger distance is not expected, considering only direct physical effects.

## 3. Discussion

One of the current challenges in radiotherapy is increasing the dose deposited specifically within the tumor, while preserving normal tissue. Theoretically, a radio-enhancement effect can be achieved by irradiating intracellular metallic nanoparticles that locally increase the tissue density and, thus, the energy absorption, producing additional secondary electrons and further increasing the ROS production and DNA damage within the cell. This was first shown in 2004 with gold nanoparticles injected in tumor-bearing mice [2]. Since then, many studies have shown the interest of this strategy, using gold, gadolinium, and hafnium particles. Besides their interest in potentializing radiotherapy, these nanoparticles are biocompatible and are also useful for concomitant RMN or X-ray imaging for theragnostics [14].

While platinum fulfills all of the physical characteristics for inducing an enhancer radiation effect, only a few studies have evaluated its potential so far. Few papers analyze the DNA breaks induced in plasmid DNA or in cell lines co-exposed to PtNPs and radiation with different beam modalities, i.e., gamma, X, or proton beams [6,7,12]. Using synthetic plasmid DNA, a net increase in DNA double breaks was observed after radiation and PtNP co-exposure, as compared to radiation alone, thus confirming the proof of concept of the enhancer effect. However, further studies analyzing the enhancer effect of PtNP in more realistic biological models, co-exposing either a model organism [4] or mouse or human cancer cell lines [3,4,5,8,9,11,15], have given rather discordant results. As also observed in the case of other nanoparticles, these discrepancies may be due to the many different parameters, such as the size, charge, and shape of NP, which could indeed be modified as soon as the NP comes in contact with biological entities. Radiation beam specificities, beam quality, and dose rates can also modulate the degree of an enhancer effect [16]. In addition, the NP uptake and localization could differ among cell types, and they are not often quantified/qualified. Finally, radiation sensitivity is greatly dependent on cell types, cell differentiation, cell proliferation, etc. [17]. Moreover, in radiobiology, at least five factors have been identified as capable of modifying the cell responses to radiation, DNA repair capacity, cell proliferation, apoptosis sensitivity, and antioxidant response [18]. Consequently, characterizing the enhancer effect by measuring a unique parameter, such as DNA breaks or cell survival, could not be sufficient to characterize the cellular response to ionizing radiation. Finally, a recent study on the radio-sensitization of in vivo models with metallic nanoparticles also highlighted a crucial contribution of the host immune system in the tumor response to combined treatments [19]. Therefore, future projects aiming at deciphering the radio-enhancement in cancer should also include in vivo models.

In the present work, we have tested the potential enhancer effect of PtNPs on two human breast cancer cell lines, MDA-MB-231 and T47D, by comparing the cellular responses in terms of cell proliferation, cell death, cell-cycle blockage, and DNA breaks and repair after exposure to gamma radiation (6 Gy). These two cell lines were chosen because they present different genetic backgrounds and a different intrinsic radiation sensitivity, as illustrated in Figure 5. This indicates that DSBs were nearly totally repaired 24 h post-exposure in the MDA-MB-231 cells, while at the same time, DSBs remained detectable in the T47D cells. In fact, with the exception of the T47D cells after a 2 Gy irradiation, we never showed any benefit of pre-exposure to PtNPs before radiation, as compared to radiation alone, in terms of cancer cell response, irrespective of the cell line and the biological endpoint. To verify that the absence of an enhancer effect was not due to a defect in the NP internalization in the cells, the NP distribution within the cell was assessed by TEM, and the intracellular PtNP was precisely quantified by ICP–MS. These two techniques clearly evidenced an internalization of the PtNPs in the two cell lines, although PtNPs enter twice as much in MDA-MB-231 (0.5 pg) as they do in T47D (0.2 pg), with a similar localization in endosomes and multivesicular bodies.

The amount of intracellular PtNPs does not seem to be limiting in terms of potentially inducing an effect, since it is of the same order of magnitude as that described by Nicol et al., who observed an enhancer effect after the exposure of MDA-MB-231 cells to gold nanoparticles [20]. In addition, using the same PtNPs, Salado-Leza et al. [8] showed a radio-enhancer effect in HeLa cells, with a larger effect using carbon ions than γ-rays. In the breast cancer cell line studied here, only a subtle effect was observed in one cell line and with a single dose. Taken together, these results demonstrate, once again, the contradictory data that can be obtained on several cell lines or biological models. One of the common hypotheses about the lack of radio-sensitization caused by metallic nanoparticles is based on the distribution of NPs within the cytoplasm exclusively. However, even in the case of a detectable enhancer effect, it has still not been shown that NPs could penetrate the nucleus, and they therefore cannot act directly on DNA. In that sense, Pagacova et al. [5] have already shown an enhancer radiation effect by using cytoplasmic PtNP, hypothesizing that PtNP caused damage to cytoplasmic structures, leading to cell death, without any detectable DNA damage.

Moreover, the localization of small metallic nanoparticles in an endosomal compartment has also been previously described, and it is commonly admitted that they end up in lysosomes, further engulfed in multivesicular bodies. PtNPs reside in a very acidic compartment, and one may wonder whether this could impair the radio-enhancer effect. This hypothesis is very unlikely, since gold NPs, with the same intracellular location, were previously shown to be beneficial in enhancing the radiation efficiency [21]. Thus, overall, the data indicate that the absence of a radiation-enhancement effect of PtNP cannot be totally attributed to a defect in the PtNPs uptake or to a specific sub-cellular localization.

Another significant point that could explain the lack of an effect of PtNP is the already described platinum antioxidant capacity, since the ROS over production within the cell is supposed to be at the origin of the radiation enhancer effect. The antioxidant properties of platinum nanoparticles have been investigated in diverse cell lines [22] and have been observed at concentrations as low as 10 µM [23]. While the concentrations used were proven to be non-toxic, the ROS were scavenged by PtNPs, with a size ranging from 2 to 5 nm. In a more recent study, Jawaid et al. [24] showed the ROS scavenging activity of PtNPs following irradiation and with a nanoparticle concentration in the range of those used in the present study. Moreover, by comparing the ROS detection after either PtNPs and irradiation or PtNPs and menadione, one can suspect that PtNPs are more prone to scavenge hydroxyl radicals than superoxide anions (Figure 5).

Given that PtNPs do not enhance the efficacy of ionizing radiation observed in this work, we performed a simulation to estimate, under our experimental conditions, the gain in terms of energy deposition and the spectra of secondary particles causing the enhancement effect that could be expected in the presence of intracellular PtNPs. If the NPs were located within the cell nucleus, then the chemical-stage simulation would allow the DNA damage produced by the interaction of ROS to be calculated. Unfortunately, in this work, where the NPs are mainly located in the lysosome, the inclusion of the chemical-stage simulation, which is now available in Geant4-DNA, will not allow any particular output useful for the interpretation of the biological results to be obtained, as it is just proportional to the increase in the energy deposition simulated here. There are currently no available data to introduce into the code that would allow for other particular aspects of chemical-stage simulation, which, in this case, could derive from the NP coating or other chemical interactions with the cell media. These considerations, together with the important computing time needed for simulating the chemical stage, drove us to exclusively study the energy deposition around the NPs and compare it with the absence of NPs.

It is important to remember that the DEF calculation method can induce a significant bias if the electronic equilibrium is not considered [13]. The three steps in which the simulation was performed, described in the material and methods section, allowed us to obtain the needed electronic equilibrium and preserve the needed statistics for the number of simulated interactions with NPs. In accordance with other published results, in our simulations, we found that the maximal DEF that can be obtained by using PtNPs, under these biological conditions (isolated NPs) and using Cesium irradiation, is around 3 in the immediate vicinity (45 nm) of the NPs. The electron spectra confirmed that the physics-related effect is localized to the very close environment of the NPs and decreases very rapidly (1.2 at 1 µM of the NPs). The question of how to interpret this extremely localized energy increase effect is nevertheless quite complex, and several parameters specific to this particular experiment, including the NP localization, size, concentration, and photon spectrum, must be taken into account.

Indeed, McMahon et al. [25] compared the increase in local energy deposition produced by gold nanoparticles with that produced in hadron therapy around the ion track. Therefore, they use the Local Effect Model [26,27], which has been developed for hadron therapy in order to calculate the equivalent Relative Biological Effectiveness (RBE) produced by those NPs in the cell. The results in this paper show an excellent agreement in the simulated survival curves with these RBE for the MDA-MB-231 cells exposed to 500 µg/mL of 1.9 nm gold nanoparticles prior to a 160 kVp X-ray exposure. However, in hadron therapy, ion tracks normally traverse the cell and often interact with the cell nucleus. Therefore, the RBE values derived in this model implicitly take into account the fact that an increase in energy deposition will occur in the cell nucleus or in other critical cell organs, which is not the case in this work, as most of the NPs are located in the lysosomes.

Other important aspects of the localized energy deposition at the nanometer scale around the NP and their biological consequences have been recently summarized in H. Rabus et al. [28]. In this paper, it is shown that the calculated DEF corresponds to a ~10 ionization in the immediate vicinity of the GNP. This important number of ionizations in 10 nm volumes, which may be responsible for coating destruction and thus a loss of biocompatibility of the NPs, may lead to their biological effect.

As already shown by Heuskin et al., who performed a GEANT4 simulation based on realistic GNP uptake and irradiations conditions [29], our model shows that the PtNP associated irradiation leads to a negligible radiation enhancement at the macroscopic level but rather an energy deposition and ROS inhomogeneities at the nanoscale. This has already been mentioned as having an important role in the way different cells react and thus in radio-sensitization [30]. Indeed, biophysical (hyperthermia, etc.) and biochemical (such as ROS) mechanisms involved in the cell response under such conditions are suggested in some studies [29] but still not well understood and therefore could not be taken into account in our model. Our results indicate that the potential enhancement strongly relies on the biological distribution of the NPs, which depends not only on the type of NP, but also on the cell type [31]; however, this distribution is not the only parameter to be taken into account in order to understand the biological effect.

## 4. Materials and Methods

### 4.1. Nanoparticles

Platinum nanoparticles of a core size of 3.2 nm were synthetized by using the radiolytic method and PEGylated, according to the method described by Li et al. [4] and Salado-Leza et al. [8]. Lyophilized PtNPs were suspended and vortexed in deionized water, reaching a 10 mM stock solution. PtNP size characterization was performed by a DLS after resuspension in water and a complete culture medium. For the cell exposure, PtNPs were dispersed in a DMEM complete medium at a 0.5 mM exposure. Taking into account the colloidal suspension and sedimentation of the nanoparticles in complex media, we maintained a constant surface concentration of 0.15 mmol/cm^2^ and always adapted the final volume of nanoparticles to the cell-culture support surface.

### 4.2. Cell Culture

Breast cancer cell lines, MDA-MB-231 (ATCC number HTB-26) and T47D (ATCC number HTB-133), were used for this study. The cells were routinely grown at 37 °C in a humidified atmosphere of 5% CO2 and 95% air in Dulbecco’s Modified Eagle’s Medium (DMEM), GlutaMAX supplemented with 10% (*v*/*v*) heat-inactivated fetal bovine serum (Sigma-Aldrich, Saint-Louis, MO, USA), and 1 mM antibiotic–antimycotic (Invitrogen, Carlsbad, CA, USA).

### 4.3. Radiation Exposure Conditions

Gamma-irradiations were performed on a GSR D1 irradiator (Gamma Medical Service, Leipzig, Germany). This self-shielded device irradiates with four sources of 137Cs, with a total activity around 180.28 TBq (measured in March 2014). The samples were irradiated at different single doses, namely 0, 2, 4, and 6 Gy, with a dose rate of 2.7 Gy/min, taking the radioactive decrease into account. The samples were irradiated in 25 cm^2^ flasks or 6- or 12-well plates. Prior to irradiation, dosimetry was performed. A cylindrical ionizing chamber 31,010 by PTW was used as the recommendation of the AAPM’S TG-61. This ionizing chamber has a cavity of 0.125 cm^3^ calibrated in 137Cs air kerma free in air at the PTB reference facility number 1904442. The polarity and the ion recombination were measured for this 137Cs source. Each measurement was corrected by the KTP factor to take the variation of temperature and atmospheric pressure into account.

### 4.4. Transmission Electron Microscopy (TEM)

MDA-MB-231 and T47D were respectively seeded at 2 × 10^5^ cells and 3 × 10^5^ in 6-well plates 24 h before the incubation. After being exposed for 2, 6, or 24 h to PtNPs, the cells were washed three times in PBS and fixed with 2% glutaraldehyde in a 0.1 M sodium cacodylate buffer (pH 7.2), for 1 h, at room temperature. The samples were then contrasted with Oolong Tea Extract (OTE) 0.5% in a cacodylate buffer, post-fixed with 1% osmium tetroxide containing 1.5% potassium cyanoferrate, gradually dehydrated in ethanol (30% to 100%), substituted gradually in a mix of ethanol–epon, and embedded in epon (Delta Microscopies, Mauressac, France). Thin sections (70 nm) were collected in 200-mesh copper grids and counterstained with lead citrate. The grids were examined with a Hitachi HT7700 electron microscope operated at 80 kV (Elexience, Verrières-le-Buisson, France), and images were acquired by using a charge-coupled device camera (AMT).

### 4.5. Mass Spectrometry (MS)

For the Mass Spectrometry, 1 × 10^6^ MDA-MB-231 and 2 × 10^6^ T47D cells were seeded in 25 cm^2^ flasks and exposed to 0.5 mM PtNPs for 2, 6, or 24 h. The cells were washed three times in PBS, before being harvested. The cells were counted in triplicate with an automatic cell counter (TC20, Biorad, Marnes la Coquette, France), before being centrifuged and digested in an HNO_3_ 16N solution. The platinum total mass was measured by using an Inductively Coupled Plasma–Mass Spectrometer (ICP–MS) and normalized to the cell number.

### 4.6. Cell Survival and Viability (Propidium Iodide Exclusion)

The cells were seeded in triplicate in 12-well plates 24 h before the PtNPs exposure. After a 6-h exposure to 0.5 mM PtNPs, cells were irradiated at 6 Gy, and the medium was changed. After 3 and 6 days, the supernatants and cells were harvested and centrifuged for 5 min at 300× *g*. The pellets were re-suspended in 500 µL of the culture medium, and the samples were further diluted to one fifth in 5 µg/mL of a propidium iodide–PBS solution. The cell survival was calculated from the volumetric absolute count, using an ACEA Novocyte flow cytometer (ACEA Biosciences, Agilent Technologies France SAS, Les Ulis, France), with an optical configuration (405, 488, and 640 nm). Live and dead cells were discriminated according to their propidium iodide exclusion, and the data were analyzed by using NovoSoftware (ACEA Biosciences, Agilent Technologies France SAS, Les Ulis, France).

### 4.7. Immunofluorescence Microscopy

The MDA-MB-231 and T47D cells were seeded at a density of 1.2 × 10^5^ and 2.5 × 10^5^ cells/well, respectively, in an 8-well glass slide and left to adhere for 48 h, before the exposure and/or irradiation. After a 6-h exposure to 0.5 mM PtNP, the cells were irradiated at 6 Gy. Then, 1, 4, or 24 h post-irradiation, the cells were washed and fixed with a 4% paraformaldehyde solution for 20 min. The cells were then permeabilized, using a PBS solution containing 0.1% Triton X-100, for 10 min. Then, the cells were stained for γ-H2A.X foci to evaluate the DNA double-strand break formation, and for Hoechst to localize nuclei. This required a blocking step using a buffer of 10% goat serum in PBS, for 1 h, at room temperature. After blocking, the cells were incubated for 75 min with mouse anti-Phospho Histone H2A.X antibody (ser139, clone JBW301, Millipore, Merck, Darmstadt, Germany) at a dilution of 1:500, in a blocking buffer. The cells were rinsed 3 times with a washing buffer (0.025% Triton X-100), before being incubated with Alexa FluorTM 488 goat anti-mouse secondary antibody (Invitrogen, ThermoFisher, Illkirch, France), at a dilution of 1:500, for 60 min, in a blocking buffer. The cells were rinsed 3 times in a washing buffer and stained with Hoechst (1 µg/mL) for 15 min at 37 °C. After a final rinse, the slides were mounted with ProlonGold anti-fade reagent (Fisher Scientific, Illkirch, France) and sealed. The foci were viewed on a spinning disk Cell observer SD microscope (Zeiss, Oberkochen, Germany). The images were treated, and the foci per cell were counted by using a cell-image analysis software (CellProfiler v4.1.3, Broad Institute of Harvard and MIT, Cambridge, MA, USA).

### 4.8. Cell-Cycle Distribution

After 3 or 6 days of culture post-exposure or irradiation, the cells were harvested and centrifuged, before re-suspension in PBS at an average concentration of 5 × 10^5^ cells/mL. The cells were fixed with a dropwise addition of 4 volumes of 70% glacial ethanol. The day after, the cells were centrifuged at 1000× *g* for 4 min and washed before staining in a 0.03 mg/mL propidium iodide and 0.05 mg/mL RNAse solution overnight. The PI fluorescence was then recorded by using a flow cytometer (Novocyte, ACEA Biosciences, Agilent Technologies France SAS, Les Ulis, France) in the PE emission canal (572 nm/28), after doublet discrimination. The cell-cycle distribution was determined by using the NovoExpress built-in cell-cycle analysis module (ACEA Biosciences, Agilent Technologies France SAS, Les Ulis, France).

### 4.9. Clonogenic Cell Survival Assay

Following a 0.5 mM PtNP exposure, the sub-confluent cells were removed from flasks, using a 0.05% of a TrypLE express solution (Fisher Scientific, Illkirch, France). The living cells were counted by using an automated cell counter (TC20, Biorad, Marnes la Coquette, France), considering trypan blue exclusion. The cells were then γ-irradiated at a dose range from 0 to 6 Gy. A colony forming assay was performed immediately after irradiation by plating cells in 60 mm-diameter Petri dishes. The MDA-MB-231 and T47D cells were left to proliferate for 14 and 28 days, respectively, and they had a plating efficiency of 40% and 30%, respectively. Then, the cells were washed and fixed in a 4% paraformaldehyde solution for 2 h. The colonies were stained with Giemsa blue (10% *v*/*v* in water) for 3 h, before extensive rinsing. The colonies containing more than 50 cells were counted under a binocular loupe, and the surviving fractions were calculated by dividing the plating efficiency in the irradiated sample by the plating efficiency in untreated conditions.

### 4.10. Oxidative Stress Measurement

The MDA-MB-231 and T47D cells were plated at a density of 4 and 8 × 10^5^ cells/well, respectively, in a 96-well plate and left to adhere for 48 h. At 6 h prior to irradiation, the medium was replaced by a complete fresh medium, with or without a 0.5 mM PtNP suspension. Menadione was used as an oxidative-stress-positive control and added in the corresponding sample at a concentration of 50 µM. Dihydroethidium (DHE) is a fluorescent probe (ex, 535 nm; em, 610 nm) for oxidative stress monitoring. Once oxidized by superoxide anion, DHE intercalates into DNA and stains the nucleus red. DHE was added at a final concentration of 10 µM, and the samples were incubated in the dark for 30 min at 37 °C. Immediately after, the plates were irradiated at 6 Gy and read at 610 nm, using a microplate reader (ClarioStarPlus, Bmg Labtech, Ortenberg, Germany). In order to get rid of any cell growth inhomogeneity, fluorescence was acquired in orbital average mode, covering the entire well. Analysis was performed by normalizing the mean fluorescence of the sample in the presence of the DHE probe, using the corresponding sample without the DHE probe.

### 4.11. GEANT4-DNA Simulation

To support the experimental observations, Monte Carlo simulations were carried out making use of the Geant4 code [32,33,34]. This general-purpose toolkit simulation, first dedicated to high-energy and nuclear physics, has been developed and improved to enable other applications in the medical or space fields. For radiobiology, some models available in Geant4-DNA [35,36,37], which are totally included in the Geant4 distribution, exist for liquid water. These models allow for the step-by-step simulation of the physical interactions of charged particles down to ~10 eV. Geant4-DNA also allows for the simulation of the diffusion and reaction of radical species resulting from the radiolysis of water.

In our simulations, only the physical aspect of the interaction of a particle with NPs has been taken into account.

The properties, in terms of energy and angular distribution, of the particles set in motion by the interactions in the NP and that escape from the NP were characterized by using Livermore models. All their energy depositions in the surrounding liquid water (approximation of a cellular medium) were calculated by using the Geant4-DNA models.

Livermore models were used in this study to model the electromagnetic processes inside the NPs, because they can be forced to describe the interactions of electrons and photons with a matter down to about 100 eV, which is adapted to our space and energy scale. New models specifically dedicated to gold nanoparticles have recently been published [38], but unfortunately, they are not yet publicly available. In the case of gold nanoparticles, a paper compared the dose estimated by either an NP-specific model or the Livermore tool. The results of this paper indicate that Livermore cross-sections overestimate the Dose-Enhancement Factor by a few percent [39].

In order to take into account the macroscopic characteristics of the cell irradiation, as well as the microscopic aspects of the interactions with single NPs and the determination of the energy deposition increase in nanometric shells around the NP, the simulations were performed in three steps.

The first step consisted of assessing the physical properties of particles (X-rays and electrons) entering a layer representing the cell culture through a detailed simulation of the whole experimental setup: an irradiator and medium culture (water, 2.2 mm thick) surrounding the cell culture (water, 10 µm thick). The primary particles were tracked inside the experimental setup, producing secondaries. All the characteristics (position, energy, and direction) of the particles entering the culture layer were recorded.

The second step consisted in injecting the particle’s characteristics recorded in the previous step (phase space file) around a nanometric volume representing the NPs in order to characterize the particles escaping from that volume. The volume was first made of water and then of Pt in order to allow for the identification of the characteristics of the spectra derived from the PtNP that could have a local effect. The NPs were represented by a spherical volume of platinum with a diameter of 3.2 nm, which corresponds to the mean diameter of NP core measured experimentally. For the present modeling, because the microscopic observations, together with the intracellular concentration, indicate that PtNPs are far from each other (more than 150 nm), we considered the PtNPs to be isolated and therefore neglected any possible secondary energy transfers.

The last step is the calculation of energy deposited by the particles obtained in the previous step. In order to calculate the Dose-Enhancement Factor (DEF), the energy deposited by these particles escaping from the NP volume is counted in concentric layers of 10 nm, which correspond to sizes comparable to sensitive molecular volumes [25,29,40,41,42] around the position of the NP in the presence of the Pt or not.

## 5. Conclusions

In this work, we analyzed different biological endpoints, such as cell survival and death, cell-cycle DNA breaks, and oxidative stress. We do not evidence any enhancer effect of PtNP in the two breast cancer cell lines, T47D and MDA-MB-231, exposed to ionizing radiation. This result is different from the one observed in the case of cervical cancer-derived (HeLa) cells [8]. The intracellular localization and concentration of PtNPs, similar in both studies, cannot explain this discrepancy.

Geant4 modeling of the early stage dose deposition in the presence of PtNPs, performed using a realistic electronic equilibrium, predicts that, at most, under our experimental conditions, the DEF could reach a maximum of 3% in the immediate vicinity of the PtNPs. The impact of this highly localized perturbation and the effect of PtNPs on the whole, particularly the contributions of the chemistry (radical chemistry, surface chemistry, and influence of molecular oxygen) and the consecutive biology, remains unclear.

Over the last decade, clinical studies have demonstrated that DEF can really be observed after a combined treatment with high-Z metallic nanoparticles and radiotherapy in certain cancer cases. This work shows that the efficacy of combined treatments is highly cancer-cell-dependent. Systematic studies are needed to evaluate various combinations of biological models. In this regard, 3D models mimicking tumors, or in vivo models, could be good alternatives.

## Data Availability

The data supporting the findings of this study are available upon request to the corresponding author.
